# Bioformulations Derived from *Enterobacter* sp. NRRU-N13 and Oligochitosan Alleviate Drought Stress in Thai Jasmine Rice (*Oryza sativa* L. var. KDML105)

**DOI:** 10.1264/jsme2.ME23025

**Published:** 2023-10-31

**Authors:** Thanakorn Saengsanga, Nutthida Phakratok, Tarntip Rattana

**Affiliations:** 1 Environmental Science Program, Faculty of Science and Technology, Nakhon Ratchasima Rajabhat University, Nakhon Ratchasima 30000, Thailand

**Keywords:** agricultural waste, antioxidant enzyme, environmental stress, PGPR, water deficit

## Abstract

Climate change is predicted to increase the length, severity, and frequency of drought, which limits plant development by changing various physiological and biochemical processes. Therefore, the present study investigated the effects of drought stress on indole-3-acetic and exopolysaccharide production by *Enterobacter* sp. NRRU-N13, developed bioformulations of plant growth-promoting *Enterobacter* sp. NRRU-N13, and evaluated the synergistic effects of these bioformulations in combination with different chitosans on the physiological responses of rice under drought stress. Drought stress inhibited the biosynthesis of indole-3-acetic and exopolysaccharides by *Enterobacter* sp. NRRU-N13. The viability and stability of *Enterobacter* sp. NRRU-N13 in bioformulations ranged between 4.70 and 5.70 log CFU g^–1^ after 80 days at an ambient temperature. Oligochitosan and chitosan at 40‍ ‍mg L^–1^ were appropriate concentrations for improving rice seedling growth, namely, plant height, root length, shoot and root fresh weights, biomass, and the vigor index (*P*<0.05). The abilities of these bioformulations, in combination with oligochitosan and chitosan, to alleviate drought stress in rice were examined. The results obtained revealed that the combined application of oligochitosan (40‍ ‍mg L^–1^) and the FON13 bioformulation (filter cake+40‍ ‍mg kg^–1^ oligochitosan+10% *Enterobacter* sp. NRRU-N13) exerted the strongest synergistic effects to alleviate drought stress in rice plants by increasing ascorbate peroxidase and catalase activities, chlo­rophyll concentrations, and relative water content and suppressing proline accumulation and electrolyte leakage from rice plants under drought stress. The present results indicate that the application of oligochitosan combined with these bioformulations effectively improved plant physiology and development. Therefore, the combined application of oligochitosan and a bioformulation of *Enterobacter* sp. NRRU-N13 is recommended to alleviate drought stress in rice plants.

Abiotic stressors, such as insufficient or excessive water, high salt, low or high temperature, and heavy metals, have a marked impact plant production and productivity and cause considerable economic losses. Abiotic stress induces oxidative stress, which increases the synthesis and accumulation of reactive oxygen species (ROS). Drought is a critical environmental concern in most countries because of its impact on plant development and production, which affects agricultural products (dos Santos *et al.*, 2022). Rice (*Oryza sativa* L.) is a crop that is essential to the global economy and serves as a staple meal for almost 50% of the world’s population. Rice cultivation necessitates a large quantity of water and, thus, rice production is significantly affected by water scarcity. In many parts of the world where rice cultivation is dependent on rain, drought is a significant issue that affects rice development and productivity (Kumar *et al.*, 2023). Thai jasmine rice cultivar KDML105 (*O. sativa* L. var. KDML105) is one of the most frequently cultivated and commercially significant varieties of rice in Thailand due to its outstanding aroma, taste, and softness. The salinity, poor fertility, and variable rainfall that plague the northeastern portion of the nation are fatal to rice farming, leading to a steep reduction in crop productivity. Therefore, it is essential to identify strategies that will reduce this risk by promoting plant growth under drought stress (Ashry *et al.*, 2022).

The application of microbial inoculants containing plant growth-promoting rhizobacteria (PGPR) has potential as an economical solution to increase crop yields by mitigating drought stress (Akhtar *et al.*, 2021; Arun *et al.*, 2020). PGPR are vital microorganisms that help plants respond more effectively to biotic and abiotic stresses. PGPR invade and occupy the surroundings or surface of roots and directly or indirectly promote plant growth and development. They accomplish this by releasing plant growth regulators or other biologically active substances, changing endogenous hormone levels, enhancing nutrient availability and uptake through fixation and mobilization, minimizing the adverse effects of pathogenic microorganisms on plants, or promoting nutrient availability and uptake. PGPR colonize the surroundings or surface of roots (Ahemad and Kibret, 2014). PGPR are also considered to be an environmentally friendly approach and sustainable green economy to alleviate drought stress and increase crop yields in multiple manners (Akhtar *et al.*, 2021). They regulate plant growth, improve soil fertility, and mitigate biotic and abiotic stresses. Several genera of PGPR of the rice rhizosphere, including *Bacillus*, *Enterobacter*, *Rhizobium*, *Ralstonia*, and *Burkholderia* have been documented (Saengsanga, 2018; Cavite *et al.*, 2021; Liu *et al.*, 2022). They induce drought tolerance by changing the structure of the host root system, osmoregulation, antioxidant defense mechanisms to scavenge ROS, hormone regulation, and the transcriptional modulation of host stress-responsive genes (Khan *et al.*, 2019). A previous study demonstrated that PGPR were an adequate immunization to increase the drought tolerance of dryland plants (Ashry *et al.*, 2022).

Chitosan (poly β-[1→4] N-acetyl-d-glucosamine) and its derivatives, such as oligochitosan, have emerged as potential replacements for synthetic pesticides because of their natural composition, antifungal activity, and capacity to activate defense systems in plant tissues [Buzón-Durán *et al.*, 2021; Narasimhamurthy *et al.*, 2022]). They are exploited in agriculture as bioformulations to promote and protect plants from biotic and abiotic stresses by triggering several sensitive genes, proteins, and secondary metabolites (Limpanavech *et al.*, 2008) as well as up-regulating ROS pathway-related genes and enhancing antioxidant defense mechanisms in plants (Singh *et al.*, 2019). Chitosan induces resistance to the osmotic stresses of rice seedlings (Pongprayoon *et al.*, 2022). They stimulate favorable responses in crops, particularly chitosan-sprayed plants, to various abiotic stressors. Additionally, chitosan boosts auxin accumulation, mainly indole-3-acetic acid (IAA), at the tip of plant roots (Lopez-Moya *et al.*, 2017).

Thailand is the second largest exporter and fourth largest producer of sugar worldwide. Sugarcane cultivation involves a large-scale factory system, resulting in a significant demand for sugarcane for sugar production and a greater need for productivity. Massive quantities of organic waste generated as byproducts of the sugar industry have been considered for composting because of the high cost of fertilizers as well as ecological sustainability. Filter cake is a byproduct that is generated during sugar production processes. It is formed when sugarcane juice is filtered to remove impurities and solid particles. The residue that remains after filtration is known as filter cake. This byproduct is generally used as an organic source for producing organic fertilizers because of its inexpensiveness, wide availability, and consistent physical and chemical properties (Mota *et al.*, 2019). It consists of 22.3% organic matter (OM), a carbon/nitrogen (C/N) ratio of 24, pH 7.7, and mineral elements, including 2.0% nitrogen (N), 1.1% phosphorous (P), 0.3% potassium (K), 2.1% calcium (Ca), 0.6% magnesium (Mg), and 0.25% sulfur (S) (Prado *et al.*, 2013), electrical conductivity (EC) of 0.80 dS m^–1^, and ash 52% (Meunchang *et al.*, 2005) on a dry weight basis. In Thailand, filter cake residue accounts for 3–9% of the sugar production process, and approximately 1.5–4.5 million tons are generated annually. Recent studies documented the use of this waste as biofertilizers and their beneficial effects on plant growth, soil biochemistry, and physical soil conditions (Mota *et al.*, 2019). The recycling of organic waste is an environmentally friendly approach that is increasingly important for managing natural resources.

Bio-inoculants are formulations that comprise beneficial microbes combined with a carrier material appropriate to their function and are simple to administer (Sohaib *et al.*, 2020). Bioformulations have been examined as an effective approach to deal with drought stress because of a number of genetic approach restrictions and expensive agricultural operations. Due to many environmental constraints, the direct application of a bacterial inoculant to the rhizosphere under natural conditions may not enhance crop growth. These ecological constraints include careless handling, which may lead to the dispersal of cells into the atmosphere or groundwater, and the short shelf life of a liquid inoculum. Therefore, some materials, known as carriers, which promote microbial proliferation and transport to the rhizosphere, are required for the successful application of an inoculant. The effectiveness of carrier-based bacterial inoculants is significantly increased by the minimal risk of contamination that they provide and their capacity to be stored for extended periods (Aloo *et al.*, 2022). Carriers are derived from an organic or inorganic origin. Some examples of organic carriers are compost, biogas slurry, crushed corn cob, charcoal, and peat (Sohaib *et al.*, 2020), while examples of inorganic carriers include perlite, lignite, zeolite, and talc. The strain of *Enterobacter* sp. NRRU-N13, isolated from the rice rhizosphere, has been shown to promote the growth of rice seedlings. Previous findings indicated that the application of this isolate exerted beneficial effects on the development of rice seedlings (Saengsanga, 2018). The cell-based bioformulation process has not yet been proven successful. Recent studies identified formulations that appear to be more reliable and consistent. Due to its many uses, the inoculation of PGPR with metabolites has potential in the development of future bioformulations (Arora and Mishra, 2016). Therefore, the present study attempted to develop bioformulations of plant growth-promoting *Enterobacter* sp. NRRU-N13 using filter cake as a carrier. The mitigating effects of the combined application of chitosan and bioformulations on the adverse effects of drought stress on rice seedlings were examined herein.

## Materials and Methods

### Rice and carrier materials

Rice (*O. sativa* L. var. KDML105), a drought- and salt-sensitive cultivar, was obtained from the Nakhon Ratchasima Rice Seed Center, Thailand.

Sugarcane filter cake was obtained from a sugar refinery in Nakhon Ratchasima Province, Thailand. Filter cake residue was air-dried to achieve a moisture content of 10%. It was black-brown with an electrical conductivity of 3.88 dS m^–1^, total dissolved solids (TDS) of 1,040‍ ‍mg L^–1^, salinity of 724 ppm, and pH 7.4.

### Effects of PEG6000 induced drought stress on IAA production by the strain

Overnight cultures were inoculated with 2% (v/v) IAA medium (g L^–1^, peptone 10, NaCl 5, yeast extract 6, and L-tryptophan 1) supplemented with 10, 20, and 30‍ ‍mg L^–1^ PEG6000 and then incubated at 30°C and 150‍ ‍rpm for 96 h. The experiment was performed in 3 replicates, with no addition of PEG6000 serving as the control. Samples were harvested after 24, 48, 72, and 96‍ ‍h and analyzed for IAA production. Briefly, cell-free supernatants were collected and mixed with 4‍ ‍mL of Salkowski’s reagent (50‍ ‍mL of 35% [v/v] HClO_4_ with 1‍ ‍mL of 0.5 M FeCl_3_) and 100‍ ‍μL of ortho-phosphoric acid. Before measurements at 535‍ ‍nm using an Evolution 600 UV-Vis Spectrophotometer, the reaction mixture was kept in the dark for 1‍ ‍h (Saengsanga, 2018). A growth curve was produced by measuring optical density (OD) at 600‍ ‍nm.

### Effects of PEG6000-induced drought stress on exopolysaccharide (EPS) production by the strain

EPS production by the strain was measured using mineral salt medium (MSM) supplemented with 10, 20, and 30‍ ‍mg L^–1^ PEG6000 and compared to that of untreated controls. MSM contained (g L^–1^) K_2_HPO_4_ 1, MgSO_4_·7H_2_O 0.5, NaCl 0.5, FeSO_4_·7H_2_O 0.001, MnSO_4_·4H_2_O 0.01, and CaCl_2_ 0.05. An overnight starter culture was prepared, inoculated, and agitated on a rotary shaker (150‍ ‍rpm) at 30°C for 72 h. Samples were taken after 24, 48, 72, and 96‍ ‍h to evaluate EPS production. The cell-free supernatant was collected for soluble EPS quantification and precipitated with two volumes of prechilled acetone. This mixture was stored at 4°C overnight before centrifugation at 12,000‍ ‍rpm and 4°C for 20‍ ‍min. Precipitated EPS was dissolved in distilled water and added to a 5% (w/v) phenol solution. Five milliliters of concentrated H_2_SO_4_ was added to the reaction mixture, and OD at 490‍ ‍nm was measured (Yadav *et al.*, 2020). The amount of EPS was calculated from a calibration curve using glucose as the standard (Dubois *et al.*, 1956).

### Screening of chitosan

To assess the effects of different chitosans and concentrations on rice growth, chitosan and oligochitosan were obtained from World Plant Chitosan and dissolved as described by Limpanavech *et al.* (2008). A stock solution (10‍ ‍g‍ ‍L^–1^) was dissolved in 2.5% (v/v) acetic acid, and the working solution was diluted in 0.01% (v/v) Triton X-100 to final concentrations of 20, 40, and 60‍ ‍mg L^–1^. Rice seeds were soaked with chitosan and oligochitosan at the given concentration for 12‍ ‍h before germination and sprayed onto 7-days-old seedlings. The germination percentage was observed 4 days after sowing (DAS). Plant height, root length, shoot and root fresh weights, and biomass were measured, and the seedling vigor index was calculated according to the following equation (Saengsanga, 2018).

Vigor index=% germination×(shoot length+root length)

### Development of filter cake-based bioformulations and viability of *Enterobacter* sp. NRRU-N13

Filter cake-based bioformulations were prepared using plant growth-promoting *Enterobacter* sp. NRRU-N13. The bacterium was re-streaked on nutrient agar (NA) that consisted of 5‍ ‍g‍ ‍L^–1^ peptone and 3‍ ‍g‍ ‍L^–1^ beef extract, and a loop full of a single colony was inoculated into 5‍ ‍mL of nutrient broth medium as a starter culture. The overnight culture was 2% (v/v) in 100‍ ‍mL of NB medium and was cultivated at 30°C for 18–24‍ ‍h on a shaker at 150‍ ‍rpm. The OD of each broth culture was adjusted to 600=1 and used as an inoculant to prepare bioformulations. Treatments were FN13 (Filter cake+10% NRRU-N13), FCN13 (Filter cake+40‍ ‍mg kg^–1^ Chitosan+10% NRRU-N13), and FON13 (Filter cake+40‍ ‍mg kg^–1^ Oligochitosan+10% NRRU-N13). In preparations, an aliquot of the bacterial suspension was aseptically transferred and mixed with sterile filter cake (10% w/v) and then adjusted to a final moisture content of 40% and kept at an ambient temperature (30–37°C) in a polyethylene bag. To provide appropriate aeration for the bioinoculants, the carrier was filled with approximately 70% of the space left vacant.

To evaluate the shelf lives of the bioformulations, the number of viable *Enterobacter* sp. NRRU-N13 in each treatment was assessed by colony-forming units (CFU), counting every 10 days for 80 days. Briefly, the bioformulation was dissolved in sterile distilled water at a ratio of 1:10 and mixed thoroughly to ensure that the microbes and carriers were separated. Ten-fold serial dilutions were performed up to 10^–5^, with the last two dilutions being spread onto NA plates. All plates were incubated at 30°C for 24 h. The number of colonies was counted and expressed as log CFU g^–1^.

### Effects of the combined application of chitosan and the bioformulation on physiological responses of rice plant

Eight different treatments were performed to assess the effects of varying chitosans and different bioformulations derived from *Enterobacter* sp. NRRU-N13 on the mitigation of drought stress in rice seedlings. Rice seeds were disinfected and germinated for 7 days. Seedlings were transplanted and grown in a pot with a 6-inch diameter containing 1‍ ‍kg of soil. The planting soil was collected from Hua Thale in Mueang Nakhon Ratchasima District, Nakhon Ratchasima Province, Thailand (14.9772N, 102.1334E), and was sterilized twice at 121°C for 15‍ ‍min. Seven days after transplanting (DAT), a fresh preparation (100 g) of each bioformulation (Control, FN13, FCN13, and FON13) and 40‍ ‍mg L^–1^ chitosan or oligochitosan were applied three times at 10, 20, and 30 DAT. After 2 days, rice seedlings were separated into two groups: 1) plants were irrigated daily to maintain a water level 2‍ ‍cm above the soil surface, and 2) plants were not irrigated to stimulate drought stress conditions for 14 days. After osmotic stress, drought-stressed plants were re-watered for 3 days. The rice plants tested were collected to assess proline accumulation, antioxidant enzyme activities, relative water content, and photosynthetic pigment.

### Measurement of proline accumulation

Rice leaves (0.25 g) were ground and dissolved in 5‍ ‍mL 3% (w/v) sulfosalicylic acid on ice. The filtrate solution was mixed with 1‍ ‍mL of ninhydrin reagent (1.25‍ ‍g ninhydrin, 30‍ ‍mL glacial acetic acid, and 20‍ ‍mL of 6 M phosphoric acid), followed by the addition of 1‍ ‍mL of acetic acid. The mixture reaction was sealed with parafilm and heated at 100°C for 1 h, and the reaction was stopped by placing the mixture on ice. The extracted solution was mixed with toluene (2‍ ‍mL). The absorbance of the resultant reaction at 520‍ ‍nm was analyzed by spectrophotometry (Evolution 600 UV-Vis Spectrophotometer). Proline concentrations were measured from a standard curve and calculated on a fresh weight basis according to the following formula (Bates *et al.*, 1973).

$$\text{Proline (µmole }\;{\text{[g fresh weight]}}^{–1}\text{)}=\frac{\text{(µg proline }{\text{mL}}^{–1}\text{)}\times \text{(amount of toluene mL)}}{\text{(115.5 µg }{\text{µmole}}^{–1}\text{)}/\text{(g sample}/5\text{)}}$$

### Assessment of antioxidant enzymes

Rice leaves (0.3 g) were separately homogenized in 1.5‍ ‍mL of ice-cold extraction buffer containing 50‍ ‍mM Tris-HCl (pH 7.8), 1‍ ‍mM EDTA, and 1.5% (w/v) polyvinylpyrrolidone. The homogenate was centrifuged at 15,000‍ ‍rpm for 20‍ ‍min. The supernatant was used as the crude extract for the enzyme activity assay.

Catalase (CAT) activity was evaluated by measuring the decrease in hydrogen peroxide (H_2_O_2_) (extinction coefficient 39.4‍ ‍Mm cm^–1^) at 240‍ ‍nm for 30‍ ‍s (Aebi, 1984). The 3-mL reaction mixture consisted of 50‍ ‍μL of a leaf extract and 15‍ ‍mM H_2_O_2_ in 100‍ ‍mM potassium phosphate buffer (pH 7.0) (Song *et al.*, 2007).

Ascorbate peroxidase (APX) activity was measured as the decrease in absorbance at 290‍ ‍nm. The reaction mixture contained 50‍ ‍mM potassium phosphate buffer (pH 7.0), 0.5‍ ‍mM ascorbate acid, 0.1‍ ‍mM H_2_O_2_, and 0.1‍ ‍mM EDTA (Nakano and Asada, 1981).

### Measurement of relative water content, electrolyte leakage, and chlo­rophyll content

To assess relative water content, rice leaves were chopped into small pieces, weighed, and scored as fresh weight. Leaf samples were soaked in distilled water in a refrigerator for 24 h, and turgid weight (TW) was measured. They were then air-dried at 72°C for 48 h, and their weight was evaluated and scored as dry weight using a 4-digit analytical balance. Relative water content was calculated using the following equation (Fariñas *et al.*, 2019).

$$\text{Relative water content}=\frac{\text{(fresh weight - dry weight)}}{\text{(turgid weight - dry weight}}\times 100$$

To assess electrolyte leakage, 100‍ ‍mg of fresh leaf and root samples were chopped into 5-mm lengths. Each sample was transferred carefully into a test tube containing 10‍ ‍mL of deionized water. Tubes were held in a water bath that was maintained at a temperature of 25°C for 15‍ ‍min. An initial measurement of electrical conductivity was performed and noted as EC1. Samples were then subjected to boiling for 15‍ ‍min followed by 25°C, and final electrical conductivity was recorded and scored as EC2. Electrolyte leakage from the leaf and root was calculated using the following equation (Dionisio-Sese and Tobita, 1998).

$$\text{Electrolyte leakage}=\frac{\text{EC1}}{\text{EC2}}\times 100$$

Photosynthetic pigments, including chlo­rophyll a (Chl *a*), chlo­rophyll b (Chl *b*), and total chlo­rophyll, were measured according to the methods of Lichtenthaler and Buschmann (2001). Briefly, 0.1‍ ‍g of fresh leaves was homogenized with 5‍ ‍mL 80% (v/v) acetone, wrapped with parafilm, and incubated in a refrigerator for 24‍ ‍h. Absorbance was measured at 663, 647, and 470‍ ‍nm using an Evolution 600 UV-Vis Spectrophotometer. Chl *a*, Chl *b*, and total chlo­rophyll were calculated using the following equations.

$$\text{Chl }\;\text{a}\text{ (µg }{\text{mL}}^{–1}\text{)}=\text{[}12.25{\text{A}}_{663}\text{)]}–\text{[}2.79{\text{A}}_{646}\text{]}$$

$$\text{Chl }\;\text{b}\text{ (µg }{\text{mL}}^{–1}\text{)}=\text{[}21.50{\text{A}}_{647}\text{]}–\text{[}5.10\;{\text{A}}_{663}\text{]}$$

$$\text{Total chlorophyll (µg }{\text{mL}}^{-1}\text{)}=\text{Chl }\text{a}+\text{Chl }\text{b}$$

### Statistical ana­lysis

Three replicates of each treatment (5 plants per replicate) were conducted using a completely randomized design (CRD). All data were analyzed and expressed as the mean±standard deviation (SD). *P*<0.05 was considered to be significant in an ana­lysis of variance (ANOVA) with the Bonferroni test.

## Results and Discussion

### Effects of PEG6000-induced drought stress on IAA pro­duction and EPS production by the strain

**Fig. 1.**
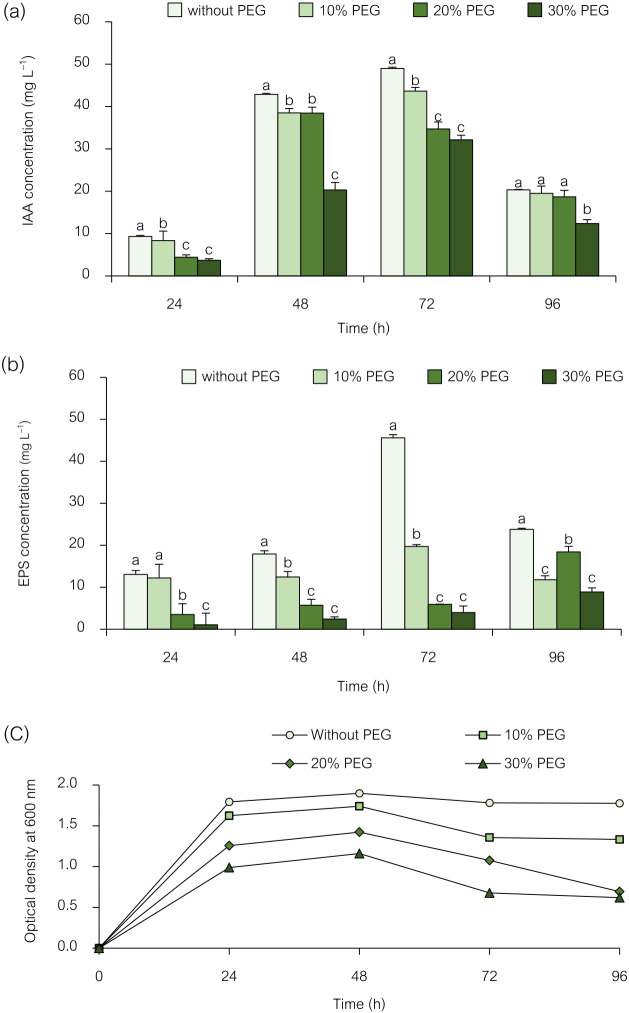
Effects of different concentrations of PEG6000 on IAA production (a), EPS production (b), and growth curve (c) of *Enterobacter* sp. NRRU-N13. Values are the mean±SD of three replicates, and different lowercase letters indicate significant differences at *P*<0.05 (Bonferroni).

Water shortage affects crop production, and drought harms rice germination and growth. IAA- and EPS-producing PGPR may accelerate drought-stressed crop growth (Abd El-Mageed *et al.*, 2022; Latif *et al.*, 2022). PGPR are considered to protect rice crops from drought. The present study investigated the effects of PEG-induced drought stress on the IAA and EPS production abilities of the isolate NRRU-N13. To achieve this, the strain was grown at different drought levels: 0, 10, 20, and 30% of PEG6000. As shown in Fig. 1, increasing the concentration of PEG6000 inhibited the growth of *Enterobacter* sp. NRRU-N13 (Fig. 1c) and the isolate synthesized the highest concentrations of IAA and EPS under non-stressed conditions. It produced varying amounts of IAA and EPS under different drought stress conditions. After 72‍ ‍h of incubation, the strains produced the highest quantity of IAA possible; this amount slightly decreased after 96 h. The highest amount of IAA (49‍ ‍mg L^–1^) was observed after 72‍ ‍h of incubation without adding PEG6000. The addition of 10, 20, and 30% PEG6000 reduced the amount of IAA to 43, 34, and 32‍ ‍mg L^–1^, respectively (Fig. 1a). As a result, a reduction in IAA production was observed when the concentration of PEG6000 was increased. Therefore, drought stress appeared to negatively affect IAA production by NRRU-N13 because PEG6000 reduces the amount of water available, thereby causing drought stress (Arun *et al.*, 2020). Previous studies confirmed that PEG-induced drought stress lowered IAA‍ ‍production in various microorganisms, including *Pseudomonas* sp. strain GAP-P45 (Sandhya *et al.*, 2009), *Bacillus endophyticus* PB3, *B. altitudinis* PB46, *B. megaterium* PB50 (Arun *et al.*, 2020), and *B. cereus* DS4 and *B. albus* DS9 (Ashry *et al.*, 2022). On the other hand, drought-tolerant *Enterobacter* sp. PAB19 produced IAA more effectively with increases in water stress (Ahmed *et al.*, 2021). However, IAA plays a crucial role in plant development and potentially mitigates the adverse effects of drought stress on rice plant growth. Therefore, an inoculation with IAA-producing bacteria has been suggested to improve the root architecture of rice and help plants cope with drought stress (Ashry *et al.*, 2022; Uzma *et al.*, 2022).

EPS production by the bacterial strain under drought stress induced by PEG6000 was assessed. As shown in Fig. 1b, increasing the concentration of PEG6000 inhibited EPS production by this strain. EPS production was detected at 24-h intervals up to 96 h. Isolate NRRU-N13 had significantly different EPS production abilities. The highest quality of EPS (45.6‍ ‍mg L^–1^) was observed at 72 h. At a 10% concentration of PEG6000, the isolate also showed the ability to produce EPS and gave 19.7‍ ‍mg L^–1^ of EPS, which decreased to 5.9 and 3.9‍ ‍mg L^–1^ at 20 and 30% PEG6000, respectively. Therefore, the bacterial strain exhibited a varying ability to produce EPS under drought-stress conditions. EPS production by bacteria involves the defense of their cells against drought by controlling nutrient and water movement across plant roots through biofilm formation, resulting in increased cell survival under stress (Morcillo and Manzanera, 2021). Several bacterial strains have been observed to produce EPS, which are a mixture of higher mole­cular weight polymers comprising carbohydrates, proteins, and other organic molecules, when exposed to stressful environments (Latif *et al.*, 2022). EPS adhere to the surfaces and aid in tying soil particles together, which improves soil aggregation.

### Screening of chitosans

To assess the effects of different types and doses of chitosans on the growth performance of rice plants, chitosan and oligochitosan were applied at concentrations of 0, 20, 40, and 60‍ ‍mg L^–1^ on rice (*O. sativa* var. KDML105) seedlings. The results demonstrated that the effects of applying chitosan and oligochitosan significantly varied for plant height and the vigor index (*P*<0.05) (Table 1). Enhancements in plant height (22.89–37.58%), root length (9.38–33.41%), shoot fresh weight (1.16–25.77%), root fresh weight (3.02–77.25%), biomass (1.50–50.25%), and the vigor index (19.35–35.55%) were observed in chitosan-treated plants. Chitosan and its derivatives have been employed as bioactive chemicals or binding materials in mixtures with other substances to promote plant growth and development (Stasińska-Jakubas and Hawrylak-Nowak, 2022) and secure protection against plant pathogens (Stanley-Raja *et al.*, 2021). The involvement of chitosan in plant defense responses to biotic and biotic stresses have been reported in several plants, such as grapes (*Vitis vinifera* L.) (Singh *et al.*, 2019) and rice (*O. sativa* L.) (Stanley-Raja *et al.*, 2021; Pongprayoon *et al.*, 2022).

Examinations of crops cultivated with chitosan have been performed to investigate the effects of different mole­cular weights and various concentrations. In one study, the application of a polymeric chitosan at 80‍ ‍mg L^–1^ by seed soaking before planting and four soil applications was found to stimu­late growth and improve rice yield (Boonlertnirun *et al.*, 2008). In the early stages of development, maize was exposed to varying concentrations of chitosan (0, 50, 75, 100, and 125‍ ‍mg L^–1^), and the maximum seed yield was recorded in maize treated with 100 and 125‍ ‍mg L^–1^ chitosan (Mondal *et al.*, 2013). Furthermore, fungal chitosan and commercial chitosan at concentrations of 25 and 50‍ ‍mg L^–1^ increased rice germination percentages (Stanley-Raja *et al.*, 2021). In the present study, oligochitosan was more effective than chitosan at each concentration, with 40‍ ‍mg L^–1^ being more effective than 20 and 60‍ ‍mg L^–1^. The strongest rice growth response, in terms of plant height, biomass, and the vigor index, was elicited by 40‍ ‍mg L^–1^ oligochitosan, followed by 40‍ ‍mg L^–1^ chitosan. This result was consistent with previous findings reported by Chamnanmanoontham *et al.* (2015), who demonstrated that oligochitosan applied at a concentration of 40‍ ‍mg L^–1^ significantly improved the vegetative growth of rice seedlings in terms of leaf and root fresh weights and dry weights. Therefore, this concentration was used in subsequent experiments.

### Viability of *Enterobacter* sp. NRRU-N13 in bioformulations

**Fig. 2.**
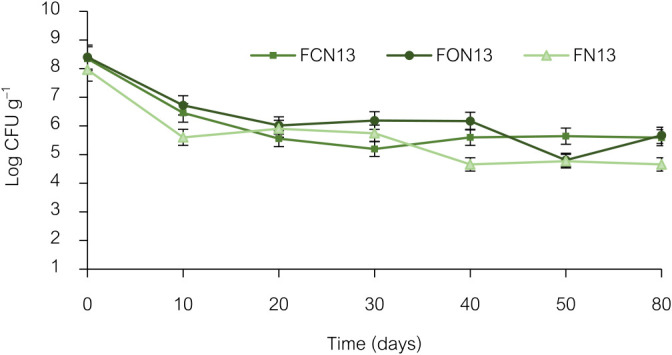
Viability of *Enterobacter* sp. NRRU-N13 with three bioformulations (FCN13, FON13, and FN13) expressed as log cfu g^–1^. Viable cell counts were performed in triplicate, and error bars represent SD.

In the present study, bioformulations of plant growth-promoting *Enterobacter* sp. NRRU-N13 delivered shelf-life stability and the potential for mitigating drought-stressed rice. Fig. 2 shows the viability of NRRU-N13 in bioformulations after 80 days of storage at an ambient temperature. The CFU counts of bacteria in all bioformulations remained the same and slightly decreased with storage progression after 10 days. At the end of the experiment, the bacterium demonstrated prolonged survival with maximum CFU counts of 5.70 log CFU g^–1^ of FON13 (Filter cake+oligochitosan+NRRU-N13), followed by FCN13 (Filter cake+chitosan+NRRU-N13), which was 5.59 CFU g^–1^. The FN13 bioformulation (Filter cake+NRRU-N13) had 4.70 log CFU g^–1^. Viability reductions in FON13, FCN13, and FN13 were 32.14, 32.97, and 40.95%, respectively, after storage at an ambient temperature for 80 days. These results suggest that bioformulations containing oligochitosan or chitosan efficiently preserved bacterial cells during storage. Oligochitosan and chitosan decreased the initial counts (10 days) of cells in bioformulations due to their natural antimicrobial capacity, but increased survival rates during storage. Many carriers, such as crushed corn cob, compost, biogas slurry, and zeolite (Sohaib *et al.*, 2020), wheat bran, rice husks, farmyard manure, bagasse, and sawdust (Aloo *et al.*, 2022), and skimmed milk, maltodextrin, sodium alginate, corn starch, and tapioca starch (Sawatphanit *et al.*, 2022), have been used to protect and maintain cell stability. However, the storage temperature is a major factor limiting bacterial viability and survival. The storage time of cells was extended by refrigeration at low temperatures. Singh *et al.* (2020) stated that talcum and sugarcane bagasse-based bioformulations developed from plant growth-promoting *Providencia vermicola* and *Klebsiella pneumonia* prolonged viability and stability under storage conditions at 4°C for 70 days. Additionally, the viability and stability of bioformulations were high when they were maintained under refrigeration (8°C) for 8 months (Aloo *et al.*, 2022). The prolonged survival of inoculants during storage is a desirable characteristic that may strengthen the industrial application of bioformulations. Bioformulations that are viable at an ambient temperature or under natural conditions may be incorporated into farming practices that lack refrigeration.

### Effects of the combined application of chitosan and bio­formulations on physiological responses of rice plants

#### Proline accumulation

**Fig. 3.**
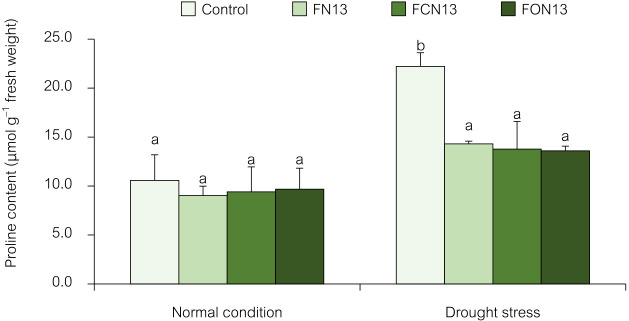
Effects of the combined application of chitosan and bioformulations on proline accumulation in rice plants. Values are the mean±SD of three replicates, and different lowercase letters indicate significant differences at *P*<0.05 (Bonferroni).

In many plants, proline accumulation is a typical response to abiotic stresses, such as salinity and drought (Chun *et al.*, 2018). The proline content in rice leaves was significantly higher in plants grown under drought stress than under normal conditions. Drought-stressed rice without the application of bioformulations had a 2-fold higher proline content than unstressed plants. Bioformulations markedly decreased proline contents in drought-stressed plants (FN13, FCN13, and FON13) (Fig. 3). However, the application of the FN13, FCN13, and FON13 bioformulations did not affect proline accumulation in rice plants under normal conditions. Previous studies reported drought-induced proline accumulation in rice leaves (Xiong *et al.*, 2012; Dien *et al.*, 2019; Pongprayoon *et al.*, 2022).

Proline plays a vital role in protecting stressed plants; therefore, the increased proline content in drought-stressed plants may be due to its adaptation to water deficiency, as reported for salt-stressed *Moringa oleifera* Lam (Elkarmout *et al.*, 2022) and drought-stressed *Allium sativum* L. (Abdelaal *et al.*, 2021). Proline is an osmoprotectant (Dien *et al.*, 2019), free radical scavenger, compatible osmolyte, cell redox balancer, enzyme protectant, subcellular structure stabilizer, and cytosolic pH buffer (Elkarmout *et al.*, 2022) to cope with drought stress. PGPR synthesize compatible solutes, antioxidants, and osmoprotectant substances that regulate osmolality in plant cells and maintain normal functions and also secrete plant growth regulators, such as IAA, to improve the root topology to uptake better resources (Akhtar *et al.*, 2021). Therefore, chitosan and NNRU-13 bioformulations may alleviate the harmful effects of drought due to their synergistic effects on proline accumulation. The beneficial effects of chitosan on stressed plants may be attributed to its involvement in elevating and regulating proline as an osmolyte and, more importantly, in maintaining plasma membrane and protein levels, as well as scavenging ROS when plants are under stress (Abdelaal *et al.*, 2021). Chitosan is also a prominent regulator of osmosis in drought stress, and since the application of chitosan led to enhanced membrane integrity and decreased lipid peroxidation in many plants, it is clear that chitosan was responsible for these positive effects (Yang *et al.*, 2009).

#### Antioxidant enzyme activities

**Fig. 4.**
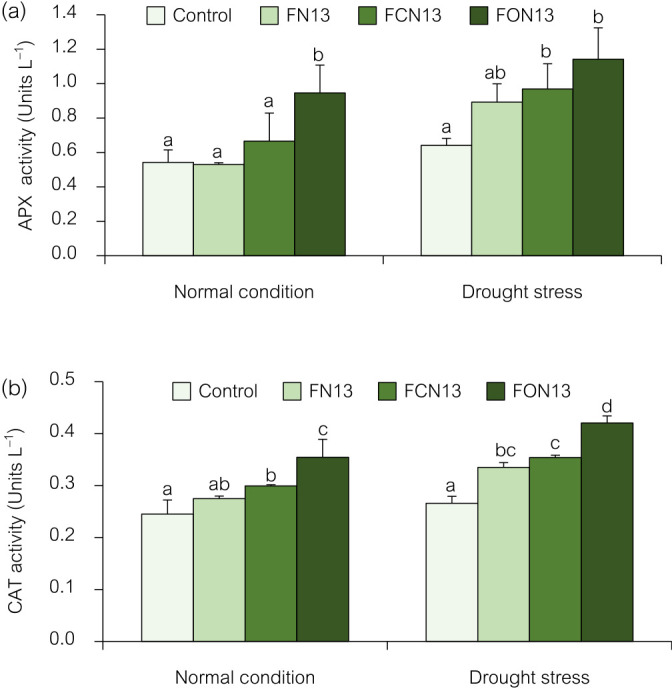
Effects of the combined application of chitosan and bioformulations on APX (a) and CAT (b) activities in rice plants. Values are the mean±SD of three replicates, and different lowercase letters indicate significant differences at *P*<0.05 (Bonferroni).

APX (EC: 1.11.1.11) and CAT (EC: 1.11.1.6) play essential roles in scavenging H_2_O_2_ when plants are exposed to stressful environmental conditions (Sofo *et al.*, 2015). APX and CAT activities were gradually decreased by the co-application of chitosan and bioformulations with and without drought stress (Fig. 4). The combined application of oligochitosan and the FON13 bioformulation induced the highest APX activity under drought stress and normal conditions of 1.14 and 0.95‍ ‍U mL^–1^, respectively, followed by chitosan+FCN13 at 0.96 and 0.66‍ ‍U mL^–1^, respectively (Fig. 4a). In the case of CAT activity, the combination of oligochitosan and the FON13 bioformulation was more efficient at inducing CAT activity in rice plants (Fig. 4b). These results demonstrated that the FCN13 and FON13 treatments significantly increased CAT activity by 0.30 and 0.35‍ ‍U‍ ‍mL^–1^, respectively, over that of the controls (0.25‍ ‍U‍ ‍mL^–1^) in rice plants under normal conditions. CAT activities were 0.33, 0.35, and 0.42‍ ‍U mL^–1^ higher under drought stress conditions with the FN13, FCN13, and FON13 treatments, respectively, than in the controls, which only exhibited 0.26‍ ‍U mL^–1^ of CAT activity. These results suggest that the application of FON13 bioformulations increased the number of antioxidant enzymes in rice and increased its drought resistance.

APX and CAT antioxidant enzymes are vital in stressed plants because they promote healthy growth while reducing oxidative damage. These effects were observed in several plants under various stressors due to an increase in ROS levels (Kamarudin *et al.*, 2018; Arun *et al.*, 2020; Abdelaal *et al.*, 2021). The application of chitosan caused changes in antioxidant enzyme activities (APX and CAT), which protected cells from drought stress. This beneficial effect of chitosan may be attributed to reductions in transpiration and the induction of stomatal closure, as well as the regulation of antioxidant enzymes, thereby mitigating the adverse effects of drought (Abdelaal *et al.*, 2021). Furthermore, PGPR may assist with the effects of drought stress. Increases in antioxidant enzymes, such as CAT, SOD, and peroxidase, were shown to be promoted by a PGPR inoculation, even when the plant was under drought stress (Hussain *et al.*, 2019). The findings of this study showed that drought stress induced the production of a large quantity of antioxidant enzymes in rice plants. Therefore, the co-application of chitosan and bioformulation applications contributes to further improvements in the activities of antioxidant enzymes, which, in turn, help to repair the damage induced by ROS (Khan *et al.*, 2019; Abdelaal *et al.*, 2021). Increases in APX and CAT activities in a stressed plant may be attributed to the application of chitosan and PGPR (Guan *et al.*, 2009). The up-regulation of these antioxidant enzymes was previously reported in chickpea (Khan *et al.*, 2019), wheat (Arun *et al.*, 2020), maize (Agbodjato *et al.*, 2021), and grape (Singh *et al.*, 2019) under abiotic stresses. These antioxidants improve the potential of a crop to overcome drought stress.

#### Chlorophyll content, relative water content, and electrolyte leakage

Since drought stress changes the physiological responses of plants, these responses need to be examined in more detail in order to gain insights into the resilience of plants and their capability to adapt to a wide variety of environmental conditions. The chlo­rophyll content, relative water content, and electrolyte leakage were assessed to clarify the effects of drought and elucidate the mechanisms by which chitosan and bioformulations assisted in ameliorating these effects.

**Fig. 5.**
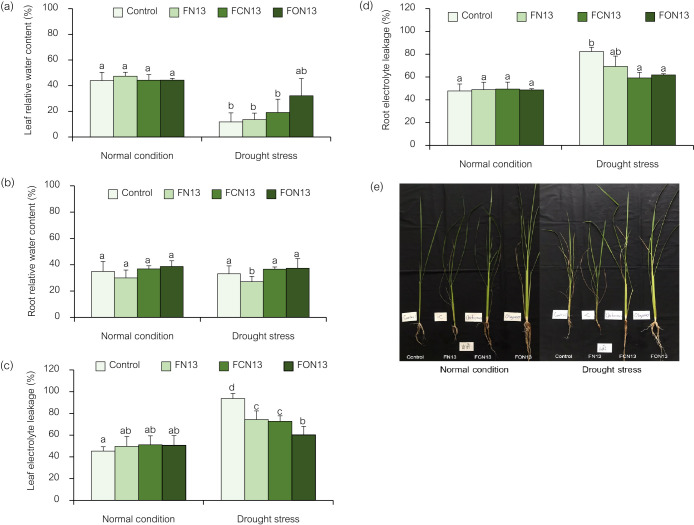
Effects of the combined application of chitosan and bioformulations on relative water contents in the leaf (a) and root (b) and electrolyte leakage from the leaf (c) and root (d) of rice plants, and responses of rice with different treatments under normal and drought stress conditions (e). Values are the mean±SD of three replicates, and different lowercase letters indicate significant differences at *P*<0.05 (Bonferroni).

The chlo­rophyll content and leaf relative water content significantly decreased in rice plants subjected to drought stress, (*P*<0.05) (Table 2 and Fig. 5). The lowest chlo­rophyll content and leaf relative water content were observed in stressed rice controls. Drought decreases the content of chlo­rophyll because it damages the chlo­rophyll pigment, degrades light-harvesting protein complexes, inhibits carbon dioxide fixation, and reduces the amount of NADP^+^ production in the Calvin cycle. Chloroplast lipids, proteins, and pigments were all oxidized under drought conditions (Moustakas *et al.*, 2022). In the present study, the total chlo­rophyll concentration was the highest in non-stressed rice treated with oligochitosan and the FON13 bioformulation. The application of chitosan+FCN13 and oligochitosan+FON13 to rice when plants were under stress conditions resulted in a significant increase in Ch *a*, Ch *b*, and total chlo­rophyll. The use of chitosan and bioformulations successfully overcame the adverse effects of drought and improved chlo­rophyll concentrations. The potential benefits of chitosan may be a consequence of increases in potassium and nitrogen, both of which are essential for healthy plant development and high yields. This may have increased not only the number of chloroplasts, but also promoted photosynthesis (Khan *et al.*, 2002).

Regarding the parameters of the present study, the root relative water content was unaffected by any treatments (Fig. 5b), whereas the leaf relative water content of rice exposed to drought significantly decreased (*P*<0.05) (Fig. 5a). Therefore, relative water content decreased from 43.91% in the non-stressed rice control to 11.77% in drought-stressed rice. This decline may be caused by the effects of drought on a plant’s capacity to absorb moisture, its permeability to humidity, and its water availability. These results are consistent with previous findings on peanut (Meher *et al.*, 2018), rice (Dien *et al.*, 2019), and garlic (Abdelaal *et al.*, 2021). However, the application of oligochitosan via spraying and the simultaneous application of the FON13 bioformulation significantly increased the relative water content by approximately 3-fold compared to the control treatments. Additionally, the combination of chitosan with the FCN13 bioformulation resulted in an approximately 2-fold increase in relative water content compared to the control treatments. The findings demonstrated that these combinations had a beneficial effect on the growth, and effectively mitigated drought-induced stress in rice plants during periods of water deficit. According to Agbodjato *et al.* (2021), the combination of chitosan and PGPR attenuated the adverse effects of drought on maize and improved yield and productivity. Moreover, it increased the chlo­rophyll content, antioxidant level, and photosynthetic pigments in drought-stressed plants (Abdelaal *et al.*, 2021).

The present study revealed that electrolyte leakage levels were higher in drought-stressed rice plants than in those not exposed to stress. Oxidative stress caused by drought has a harmful impact on plasma membranes and permeability; therefore, this increase may be associated with these changes (Abdelaal *et al.*, 2021). Nevertheless, leaf electrolyte leakage decreased from 93.67% in drought-stressed rice to 60.23% in rice treated with oligochitosan and the FON13 bioformulation (Fig. 5c). Electrolyte leakage detected following the application of chitosan and the FCN13 bioformulation was 72.74%. These treatments against electrolyte leakage were more successful when used simultaneously. Root electrolyte leakage slightly decreased in treated rice plants (Fig. 5d). Drought has been suggested to induce abnormalities in the interaction between membrane lipids and proteins, thereby affecting membranes’ ability to transport substances across their surfaces (Ansari *et al.*, 2021). The concurrent application of oligochitosan or chitosan with bioformulations maintained membrane stability and decreased electrolyte leakage. Abdelaal *et al.* (2021) reported similar findings, indicating that the application of chitosan significantly decreased electrolyte leakage. Furthermore, PGPR has been shown to reduce electrolyte leakage in drought-stressed plants (Saleem *et al.*, 2021). Synergistic combinations may improve drought tolerance in rice plants by reducing proline accumulation and electrolyte leakage while concurrently increasing the activities of antioxidant enzymes as well as chlo­rophyll and relative water contents. Therefore, the concurrent application of oligochitosan and *Enterobacter* sp. NRRU-N13 bioformulations may mitigate the detrimental effects of drought stress.

## Conclusion

Based on the present results, drought stress induced by PEG6000 exerted detrimental effects on the biosynthesis of IAA and EPS by *Enterobacter* sp. NRRU-N13. IAA and EPS levels decreased as a direct consequence of an increase in the concentration of PEG6000. Drought stress induced physiological modifications in Thai jasmine rice (*O. sativa* L. var. KDML105). In the majority of parameters tested, the combination of chitosan and rhizobacteria was superior to chitosan alone. The bioformulation of *Enterobacter* sp. NRRU-N13 and the concurrent application of oligochitosan effectively alleviated the impact of drought stress on rice plants. This synergistic combination promises to enhance rice drought tolerance by reducing proline accumulation and electrolyte leakage while simultaneously increasing antioxidant enzyme activity, the concentration of chlo­rophyll, and relative water content. Therefore, the combination of oligochitosan and *Enterobacter* sp. NRRU-N13 bioformulations has potential as a biostimulant to improve rice development during drought stress.

## Citation

Saengsanga, T., Phakratok, N., and Rattana, T. (2023) Bioformulations Derived from *Enterobacter* sp. NRRU-N13 and Oligochitosan Alleviate Drought Stress in Thai Jasmine Rice (*Oryza sativa* L. var. KDML105). *Microbes Environ ***38**: ME23025.

https://doi.org/10.1264/jsme2.ME23025

## Figures and Tables

**Table 1.** Effects of different chitosan treatments on the growth performance of germinated rice seedlings

Treatment	Concentration (mg L^–1^)	Germination percentage	Plant height (cm)	Root length (cm)	Shoot fresh weight (mg 5 plants^–1^)	Root fresh weight (mg 5 plants^–1^)	Biomass (mg 5 plants^–1^)	Vigor index
Control	0	100.00	4.63±0.43^a^	4.88±0.46^a^	15.52±2.34^a^	15.87±1.58^a^	5.99±0.19^a^	936.4±68^a^
Chitosan	20	100.00	5.69±0.68^b^	4.92±1.02^a^	17.85±0.63^a^	27.18±4.26^b^	6.08±0.01^ab^	1061±57^b^
			(22.89%)	(12.59%)	(15.01%)	(71.27%)	(1.50%)	(19.35%)
	40	100.00	5.82±0.94^b^	5.60±0.75^b^	19.12±0.32^a^	23.15±9.91^ab^	6.86±0.37^ab^	1142±68^bc^
			(25.70%)	(28.15%)	(23.20%)	(45.87%)	(14.52%)	(28.46%)
	60	100.00	6.03±0.41^b^	4.91±1.03^a^	18.74±0.32^a^	16.35±3.45^a^	7.10±0.27^abc^	1094±96^bc^
			(30.24%)	(12.36%)	(20.75%)	(3.02%)	(18.53%)	(23.06%)
Oligochitosan	20	100.00	5.84±0.34^b^	4.88±0.33^a^	19.52±1.04^a^	22.89±1.39^ab^	8.17±2.17^bc^	1072±59^b^
			(26.13%)	(11.67%)	(25.77%)	(44.23%)	(36.39%)	(20.58%)
	40	100.00	6.37±0.11^b^	5.83±0.51^b^	18.92±0.98^a^	28.13±1.67^b^	9.00±1.68^c^	1205±68^c^
			(37.58%)	(33.41%)	(21.91%)	(77.25%)	(50.25%)	(35.55%)
	60	100.00	6.08±0.60^b^	4.78±0.59^a^	15.77±2.22^a^	21.32±4.58a^b^	7.74±0.84^abc^	1073±99^b^
			(31.32%)	(9.38%)	(1.16%)	(34.34%)	(29.22%)	(20.70%)

Values are the mean±SD of three replicates (5 plants per replicate), with lowercase letters indicating significant differences at *P*<0.05 (Bonferroni). Values in parentheses indicate the average percentage increase relative to the control.

**Table 2.** Effects of the combined application of chitosan and bioformulations on chlo­rophyll contents in rice plants

Conditions	Treatments	Chlorophyll contents (μg g^–1^ fresh weight)		
		Ch *a*	Ch *b*	Total chlo­rophyll
Normal condition	Control	17.20±6.54^ab^	12.38±2.97^a^	29.59±3.57^ab^
	FN13	21.90±0.23^a^	16.33±4.03^a^	38.23±3.84^a^
	FCN13	19.63±2.11^a^	17.57±3.90^a^	37.20±1.97^a^
	FON13	21.1±0.74^a^	22.31±1.99^a^	43.39±2.48^c^
Drought-stressed condition	Control	12.11±2.27^b^	7.02±3.37^b^	19.14±2.23^b^
	FN13	19.70±2.65^a^	12.08±5.98^a^	25.05±6.50^b^
	FCN13	19.98±0.77^b^	18.81±3.79^a^	38.79±4.49^a^
	FON13	19.61±1.70^a^	17.20±6.50^a^	36.82±7.39^ab^

Values are the mean±SD of three replicates and different lowercase letters indicate significant differences at *P*<0.05 (Bonferroni).
